# Spotlight on the Energy Harvest of Electroactive Microorganisms: The Impact of the Applied Anode Potential

**DOI:** 10.3389/fmicb.2019.01352

**Published:** 2019-06-26

**Authors:** Benjamin Korth, Falk Harnisch

**Affiliations:** Department of Environmental Microbiology, Helmholtz Centre for Environmental Research - UFZ, Leipzig, Germany

**Keywords:** electroactive microorganisms, extracellular electron transfer, microbial thermodynamics, microbial energy harvest, electron-transport chain, modeling

## Abstract

Electroactive microorganisms (EAM) harvest energy by reducing insoluble terminal electron acceptors (TEA) including electrodes via extracellular electron transfer (EET). Therefore, compared to microorganisms respiring soluble TEA, an adapted approach is required for thermodynamic analyses. In EAM, the thermodynamic frame (i.e., maximum available energy) is restricted as only a share of the energy difference between electron donor and TEA is exploited via the electron-transport chain to generate proton-motive force being subsequently utilized for ATP synthesis. However, according to a common misconception, the anode potential is suggested to co-determine the thermodynamic frame of EAM. By comparing the model organism *Geobacter* spp. and microorganisms respiring soluble TEA, we reason that a considerable part of the electron-transport chain of EAM performing direct EET does not contribute to the build-up of proton-motive force and thus, the anode potential does not co-determine the thermodynamic frame. Furthermore, using a modeling platform demonstrates that the influence of anode potential on energy harvest is solely a kinetic effect. When facing low anode potentials, NADH is accumulating due to a slow direct EET rate leading to a restricted exploitation of the thermodynamic frame. For anode potentials ≥ 0.2 V (vs. SHE), EET kinetics, NAD^+^/NADH ratio as well as exploitation of the thermodynamic frame are maximized, and a further potential increase does not result in higher energy harvest. Considering the limited influence of the anode potential on energy harvest of EAM is a prerequisite to improve thermodynamic analyses, microbial resource mining, and to transfer microbial electrochemical technologies (MET) into practice.

## Introduction

According to textbook knowledge, respiring microorganisms harvest energy by coupling the oxidation of a soluble electron donor (i.e., the substrate) to the reduction of a soluble terminal electron acceptor (TEA). This process is termed catabolism and allows microorganisms, more specifically chemotrophs, to harvest energy for, *inter alia*, anabolic processes. Thereby, electrons of the electron donor are released from a higher energy level to the acceptor and the energetic difference drives the reaction. This is reflected by the difference in electrochemical potential, *E*, or its equivalent Gibbs free energy, Δ*G*, of the redox-pair (Equation 1)^1^.

(1) $$\begin{array}{r} E=\frac{-\Delta G}{zF} \end{array}$$

More particular, electrons are transferred from the electron donor to intracellular electron carriers (e.g., NAD^+^), subsequently further to the electron-transport chain (ETC) and finally to the TEA. Microorganisms can obtain different amounts of energy from the oxidation of the same substrate by utilizing different soluble TEA depending on the respective redox potential (e.g., O_2_ > N${\text{O}}_{3}^{-}$ > S${\text{O}}_{4}^{2-}$). Electroactive microorganisms (EAM) perform a unique strategy for harvesting energy called extracellular electron transfer (EET). During EET, a protein network transfers electrons across the cell membrane to insoluble TEA (Lovley, 2012).

EET attracted considerable attention during the last years. This interest is driven by the potential role EET plays in natural redox-cycles as well as the promise for its exploitation in technical systems when EAM are interfaced to electrodes (Rabaey et al., 2009; Koch and Harnisch, 2016). These primary microbial electrochemical technologies (MET) are envisaged to be used for, e.g., cleaning of wastewater, electric power production, and synthesis of chemicals (Logan and Rabaey, 2012; Schröder et al., 2015).

A whole arsenal of techniques have been applied for shedding light on the fundamentals of EET and EAM. These include, for instance, cyclic voltammetry, confocal resonance Raman microscopy, differential electrochemical mass spectrometry, nuclear magnetic resonance spectroscopy, and optical coherence tomography (Fricke et al., 2008; Virdis et al., 2012; Alves et al., 2015; Kubannek et al., 2018; Molenaar et al., 2018). However, a comprehensive thermodynamic analysis of the energy fluxes during EET is still missing. As thermodynamics describes the efficiencies of energy conversions and thereby the likelihood of processes to occur, its understanding is a basic prerequisite for deciphering the role of EAM in nature as well as for transferring MET from lab scale to industrial applications (Von Stockar, 2010, 2013; Sadhukhan et al., 2016). Certainly, also genetics, the proteome, and reaction kinetics co-determine microbial activity. Nevertheless, the microbial window of opportunities is firstly defined by thermodynamics and its utilization by the other factors or in metaphoric terms: “*Thermodynamics sets the frame, evolution draws the picture*” (Schoepp-Cothenet et al., 2013). So far, energetic assessments of EAM were each restricted to single aspects of thermodynamics: either quantification of biomass production, Gibbs free energy calculations (only valid with corrections for non-standard conditions), or measuring of heat production (Mahadevan et al., 2006; Schröder, 2007; Marsili et al., 2010; Korth et al., 2015, 2016). For an overall thermodynamic analysis, these isolated aspects need to be linked and measured in the same condition (in best case scenario in one set of experiments). Only thereby, a comprehensive energy balance for EAM under realistic experimental conditions can be generated. Mastering this challenging task will lead to a progressed understanding of the ecological role of EAM and increase the feasibility for applications.

For establishing a solid foundation for future research in thermodynamics of EAM, thermodynamic calculations and their adaption for considering direct EET are described within this article. Moreover, by discussing bioenergetic fundamentals and modeling energy harvest of EAM, the occasionally encountered misconception that the anode potential co-determine the thermodynamic frame is refuted.

## Thermodynamic Calculations on Microorganisms Using Soluble Terminal Electron Acceptors

Catabolic energy harvest of microorganisms is assessed by calculating the energy difference between oxidation of electron donor (i.e., anodic reaction) and reduction of electron acceptor (i.e., cathodic reaction). This principle is exemplified for the anaerobic oxidation of acetate (Equation 2) coupled to the reduction of nitrate to nitrite (Equation 3).

(2) $$\begin{array}{r} \text{C}{\text{H}}_{3}\text{CO}{\text{O}}^{-}+4{\text{H}}_{2}\text{O}\to {2\text{HCO}}_{3}^{-}+{9\text{H}}^{+}+{8\text{e}}^{-} \end{array}$$

(3) $$\begin{array}{r} {\text{NO}}_{3}^{-}+2{\text{H}}^{+}+2{\text{e}}^{-}\to {\text{NO}}_{2}^{-}+{\text{H}}_{2}\text{O} \end{array}$$

By considering the number of released/consumed electrons, Equations 1 and 2 are combined yielding the catabolic reaction (Equation 4).

(4) $$\begin{array}{r} {\text{CH}}_{3}\text{CO}{\text{O}}^{-}+4\text{N}{\text{O}}_{3}^{-}\to {2\text{HCO}}_{3}^{-}+4\text{N}{\text{O}}_{2}^{-}+{\text{H}}^{+} \end{array}$$

The reaction stoichiometry and tabulated Gibbs free energies of formation ($\Delta_{\text{f}}G^{0^{\prime }}$) are used for calculating Gibbs free energy of catabolic reaction ($\Delta_{\text{R}}G_{\text{cat}}^{0^{\prime }}$, Equation 5). The obtained value represents the microbial energy harvest from coupling acetate oxidation with reduction of nitrate for biochemical standard conditions^2^.

(5) $$\begin{array}{l} \Delta_{\text{R}}G_{\text{cat}}^{0^{\prime }}\text{=}\Delta_{\text{f}}G^{0^{\prime }}\left({\text{2HCO}}_{\text{3}}^{-}\text{+ }{\text{4NO}}_{\text{2}}^{-}\text{+}{\text{H}}^{\text{+}}-\;{\text{CH}}_{\text{3}}{\text{COO}}^{-}-\;{\text{4NO}}_{\text{3}}^{-}\right)\\ \text{=}[\text{2}\left(-\text{586}.\text{9}\right)\text{+ 4}\left(-\text{37}.\text{2}\right)\text{+}\left(-\text{39}.\text{9}\right)\\ -\left(-\text{369}.\text{4}\right)-\text{4}\left(-\text{111}.\text{3}\right)]\frac{\text{kJ}}{\text{mol}}\text{=}-\text{547}.\text{9}\frac{\text{kJ}}{\text{mol}} \end{array}$$

For obtaining realistic Gibbs free energies of catabolic reactions (Δ_R_*G*_cat_) allowing a reasonable assessment of the process, a correction for non-standard conditions (i.e., actual reaction conditions) is indispensable (Equation 6)^3^. The correction is generally done for concentrations of reactants and can be further improved by including temperature via the van 't Hoff equation (Heijnen and Kleerebezem, 2010).

(6) $$\begin{array}{l} \Delta_{\text{R}}G_{\text{cat}}=\Delta_{\text{R}}G_{\text{cat}}^{0^{\prime }}+RT\;\ln[{\left(\frac{C_{\text{HCO3}-}}{C_{\text{HCO3}-}^{0^{\prime }}}\right)}^{2}{\left(\frac{C_{\text{NO2}-}}{C_{\text{NO2}-}^{0^{\prime }}}\right)}^{4}\\ \;\left(\frac{C_{\text{H+}}}{C_{\text{H+}}^{0^{\prime }}}\right)\left(\frac{C_{\text{Ac}-}^{0^{\prime }}}{C_{\text{Ac}-}}\right){\left(\frac{C_{\text{NO3}-}^{0^{\prime }}}{C_{\text{NO3}-}}\right)}^{4}] \end{array}$$

## Transferring Thermodynamic Principles to Electroactive Microorganisms

For illustrating thermodynamic calculations on EAM, the model organism *Geobacter* spp. is assumed coupling the oxidation of acetate with direct EET to an anode with a fixed potential. The electron transfer to the anode represents the reduction reaction, and thus the anode potential is occasionally used for calculations of catabolic energy harvest. Consequently, the anode potential would co-determine the thermodynamic frame (i.e., maximum available energy from substrate oxidation) as the energy harvest is based on the energy difference between electron donor and TEA (here anode). In our opinion, this approach is not valid and the assumption that the anode potential defines the energy level of the reduction reaction is a misconception. As outlined in the next section and according to fundamental principles of biological energy conversion, energy levels of intracellular electron carriers should be used for conducting thermodynamic calculations resulting in a decreased thermodynamic frame of EAM when direct EET is performed.

## Comparing the Electron-Transport Chains of Electroactive Microorganisms and Microorganisms Respiring Soluble Terminal Electron Acceptors

**Figure 1:**
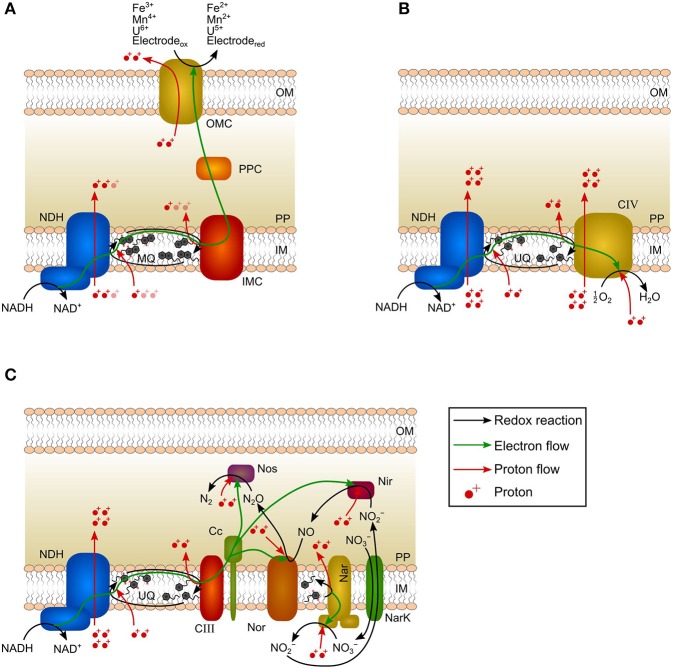
Schematic canonical electron-transport chains of *Geobacter* species, *Escherichia coli*, and *Paracoccus denitrificans*. **(A)** In *Geobacter* spp., two electrons per NADH are transferred to a TEA via NADH dehydrogenase (NDH), menaquinone pool (MQ), inner membrane cytochrome (IMC), periplasmic cytochrome (PPC), and outer membrane cytochrome (OMC). The generated proton-motive force (*pmf*) is 1.5–3 H^+^/e^−^. Pale-colored protons indicate current uncertainties in the generated *pmf*. The redox-Bohr effect leads to the translocation of 1 proton per transferred electron to the extracellular space. **(B)** In *E. coli*, two electrons per NADH are transferred to oxygen via NADH dehydrogenase (NDH), ubiquinone pool (UQ), and complex IV (CIV). All reactions occur at the inner membrane resulting in a *pmf* of 5 H^+^/e^−^. **(C)** In *P. denitrificans*, two electrons per NADH are transferred via NADH dehydrogenase (NDH), ubiquinone pool (UQ), complex III (CIII), cytochrome *c* (Cc), nitrate reductase (Nar), nitrite reductase (Nir), nitric oxide reductase (Nor), and nitrous oxide reductase (Nos) to nitrogen. NarK: nitrate/nitrite transporter. Nir, Nor, and Nos consume protons from periplasm. The generated *pmf* is 3 H^+^/e^−^. OM: outer membrane; PP: periplasm; IM: inner membrane.

The ETC is the core of microbial energy harvest. It converts catabolically derived reducing equivalents (e.g., the intracellular two-electron carrier NADH) into proton-motive force (*pmf* , i.e., proton gradient across the inner membrane) being subsequently utilized for ATP-synthesis (Schoepp-Cothenet et al., 2013). In *Geobacter* spp., NADH is oxidized within cytoplasm by an inner membrane-bound NADH-dehydrogenase and electrons are further transferred via menaquinones and inner membrane cytochromes (e.g., MacA) to periplasmic cytochromes (e.g., PpcA-E) (Figure 1A; Kracke et al., 2015; Santos et al., 2015). Afterward, electrons are transferred to outer membrane cytochromes that are specific for the TEA (Levar et al., 2012). When performing direct EET, the NADH-dehydrogenase is described to translocate 1–1.5 protons per transferred electron (H^+^/e^−^) to the periplasm (Figure 1A; Champine et al., 2000; Mahadevan et al., 2006; Feist et al., 2014). Model approaches indicate that oxidation of menaquinones by inner membrane or periplasmic cytochromes provides additional *pmf* with a stoichiometry of 0.5–1.5 H^+^/e^−^ (Mahadevan et al., 2006; Feist et al., 2014). Subsequent electron transfer steps within the periplasm and across the outer membrane do not create *pmf* as the respective cytochromes cannot facilitate proton translocation across the inner membrane. This was already shown for the EAM model organism *Shewanella oneidensis* (Tokunou et al., 2015; Okamoto et al., 2017). Thus, ETC of *Geobacter* spp. is characterized by a H^+^/e^−^ ratio of 1.5–3 that is only exploiting the energy difference between NADH and inner membrane cytochromes (Figure 1A). The energy difference between inner membrane cytochromes and TEA (anode) is dissipated.

Furthermore, charge balancing requires the transport of one proton across the outer membrane for every electron being transferred by direct EET to the TEA (Figure 1A). This is accomplished by outer membrane cytochromes and the ability of cytochromes to act as proton pump is referred to as redox-Bohr effect (Morgado et al., 2012). This proton translocation can theoretically lead to the formation of a proton gradient across the outer membrane that could be seized for ATP generation. However, this would require the existence of an outer-membrane ATP-synthase that is so far not described for gram-negative bacteria and rather represents a relic of evolution (Küper et al., 2009; Nicholls and Ferguson, 2013). Conversely, the redox-Bohr effect rather diminishes the *pmf*.

In contrast to EAM, all reactions of the ETC of the model chemotroph *Escherichia coli* respiring oxygen as TEA occur at the inner membrane. In a typical scenario, electrons are transferred via NADH-dehydrogenase and ubiquinones to an oxygen reducing cytochrome resulting in the transport of 5 H^+^/e^−^ (Figure 1B; Puustinen et al., 1989; Bogachev et al., 1996; Unden and Bongaerts, 1997; Chen and Strous, 2013). The ETC of *E. coli* is versatile and the H^+^/e^−^ ratio varies depending on the availability of electron donor and electron acceptor (Unden and Bongaerts, 1997). As all steps of the ETC occur at the inner membrane, the energy difference between the reduction equivalent NADH and TEA can be fully exploited (Figure 1B).

Nitrate-respiring microorganisms (e.g., *Paracoccus denitrificans*) are comparable to EAM since a considerable share of ETC reactions also occur beyond the inner membrane (Figure 1C; Berks et al., 1995; Shapleigh, 2006; Hino et al., 2012; Olaya-Abril et al., 2018). The NADH dehydrogenase and ubiquinones are followed by several NOx reductases in order to reduce nitrate to dinitrogen (Figure 1C; Chen and Strous, 2013). Due to the localization of reductases, only NADH dehydrogenase, ubiquinones, and nitrate reductase contribute to the generation of *pmf* (Figure 1C; Bertero et al., 2003; Chen and Strous, 2013). Consequently, only 3 H^+^/e^−^ are transported to the periplasm during nitrate reduction to dinitrogen, although the TEA is reduced intracellularly and the last reduction step has a higher redox potential (N_2_O/N_2_, *E*^0′^ = 1.36 V)^4^ compared to the final reduction step in the ETC of oxygen-respiring microorganisms (H_2_O/O_2_, *E*^0′^ = 0.82 V) (Chen and Strous, 2013).

In bioenergetics, it is generally agreed that periplasm and outer membrane are not energized compartments (i.e., proteins localized there do not generate *pmf*) (Nicholls and Ferguson, 2013). Based on this consensus and the comparison of EAM with nitrate-respiring microorganisms (several reaction steps of the ETC occur within the periplasm resulting in similar H^+^/e^−^ ratios), it can be concluded that the generation of *pmf* in EAM is restricted to reactions at the inner membrane and further electron transfer reactions (within periplasm and across the outer membrane) do not contribute to energy harvest. As a clear consequence, the electron transfer to the anode can also not contribute to the energy harvest. Hence, a change in anode potential does not necessarily have an impact on the energy harvest.

As deduced above, it is not correct to calculate the energy harvest of EAM by using the energy difference between substrate and anode. This assumption overestimates the microbial energy harvest and leads to a non-existent, immanent correlation to the applied anode potential. Moreover, this approach neglects fundamentals of biological energy conversion (i.e., the generation of *pmf*) and physiology of direct EET (i.e., a considerable share of ETC proteins is located within the periplasm and at the outer membrane). Instead, the thermodynamic frame of the energy harvest of EAM has to be assessed by calculating the energy difference between substrate and an intracellular electron carrier (e.g., NAD^+^/NADH) or an inner membrane cytochrome (Bird et al., 2011) as described in the next section.

## Modeling the Energy Harvest of Electroactive Microorganisms

**Figure 2:**
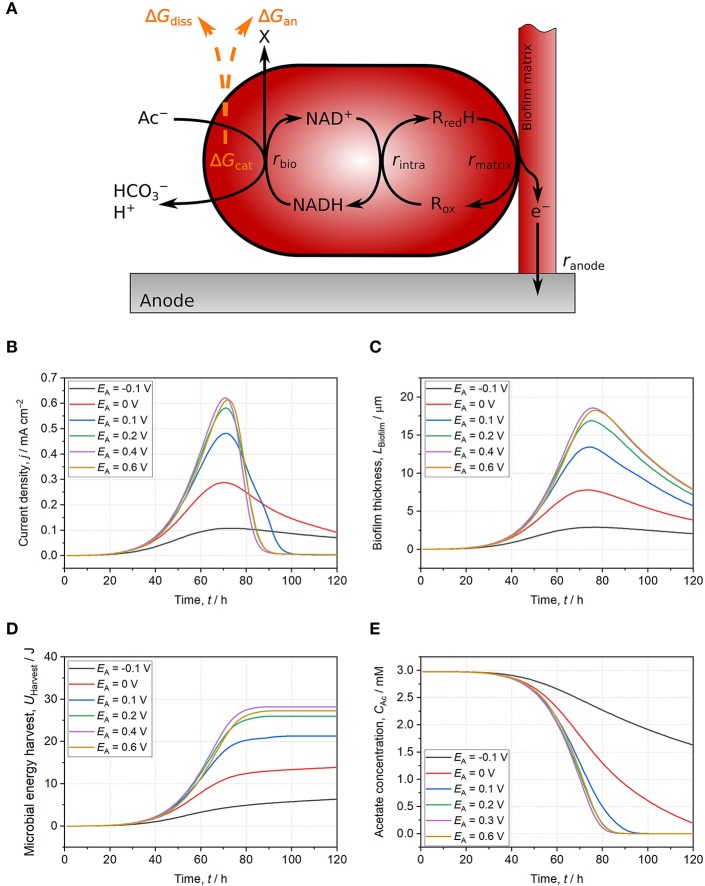
Schematic illustration of the used model and model results for *Geobacter* spp. biofilms growing on anodes set to −0.1 V (black line), 0 V (red line), 0.1 V (blue line), 0.2 V (green line), 0.4 V (purple line), and 0.6 V (yellow line). **(A)** Schematic model representation: Acetate oxidation is coupled to NAD^+^ reduction resulting in energy harvest (Δ*G*_cat_) subsequently used for the build-up of biomass (Δ*G*_an_) and for providing driving force for growth (Δ*G*_diss_). Electrons are then transferred to intracellular cytochromes and further to a conductive biofilm matrix. Finally, electrons are donated to the anode. All reactions occur at individually calculated rates (*r*_bio_, *r*_intra_, *r*_matrix_, *r*_anode_) (Korth et al., 2015). **(B)** Current density. **(C)** Biofilm thickness. **(D)** Microbial energy harvest. **(E)** Acetate concentration. With anode potentials ≤ 0.1 V, the thermodynamic frame defined by acetate, NAD^+^/NADH ratio, and other reactants is not fully exploited. Slow EET kinetics result in thermodynamically unfavorable reaction conditions for catabolic reaction (i.e., low NAD^+^/NADH ratio) leading to lower current density, biofilm thickness, and microbial energy harvest at comparable acetate consumption. For anode potentials ≥ 0.2 V, direct EET is not limiting catabolism and reaction conditions are thermodynamically improved. Consequently, current density, biofilm thickness, and microbial energy harvest do not further increase with higher potentials. The model is based on separated anodic compartment (volume = 250 mL, anode area = 10 cm^2^) and cathodic compartment via a membrane (membrane area = 10 cm^2^). Acetate concentration is 3 mM, phosphate buffer concentration is 50 mM, and initial pH is 6.95. Further model parameters are detailed in Supplementary Table S1 and Korth et al. (2015).

The Gibbs free energy balance of EAM performing direct EET including catabolic and anabolic reactions, energy dissipation, maintenance as well as growth and electrochemical performance can be modeled (Figure 2A; Korth et al., 2015). This model framework is applied in the following to assess the influence of anode potential on energy harvest and growth of *Geobacter* spp. biofilm. In the model, acetate oxidation is coupled to NAD^+^ reduction. Subsequently, electrons are transferred to intracellular cytochromes and further to a conductive biofilm matrix (Figure 2A). Direct EET to the anode at a set potential (*E*_A_) is performed by cytochromes within the biofilm matrix with a formal potential of *E*^f^ = −0.136 V (Fricke et al., 2008). Main model parameters are listed in the Supplementary Table S1 and further details can be found in Korth et al. (2015). As many molecular details of the energy conversion of microorganisms using insoluble TEA are unknown (Bird et al., 2011), the energy-yielding processes in the model were simplified to one catabolic reaction: acetate oxidation coupled to NAD^+^ reduction (Equation 7). Gibbs free energy of this catabolic reaction is calculated and corrected for non-standard conditions according to Equations 8 & 9.

(7) $${\text{CH}}_{\text{3}}{\text{COO}}^{-}\text{+ }{\text{4H}}_{\text{2}}\text{O }{\text{+ 4NAD}}^{\text{+}}\to {\text{2HCO}}_{\text{3}}^{-}{\text{+5H}}^{\text{+}}\text{+ 4NADH}$$

(8) $$\begin{array}{l} \Delta_{\text{R}}G_{\text{cat}}^{0^{\prime }}=\\ \Delta_{\text{f}}G^{0^{\prime }}\left({\text{2HCO}}_{\text{3}}^{-}\text{+ }{\text{5H}}^{\text{+}}\text{+ 4NADH}-{\text{CH}}_{\text{3}}{\text{COO}}^{-}-{\text{4H}}_{\text{2}}\text{O}-{\text{4NAD}}^{\text{+}}\right) \end{array}$$

(9) $$\begin{array}{l} \Delta_{\text{R}}G_{\text{cat}}=\\ \Delta_{\text{R}}G_{\text{cat}}^{0^{\prime }}+RT\;\ln\left[{\left(\frac{C_{\text{HCO3}-}}{C_{\text{HCO3}-}^{0^{\prime }}}\right)}^{2}{\left(\frac{C_{\text{H+}}}{C_{\text{H+}}^{0^{\prime }}}\right)}^{5}{\left(\frac{C_{\text{NADH}}}{C_{\text{NAD+}}}\right)}^{4}\left(\frac{C_{\text{Ac}-}^{0^{\prime }}}{C_{\text{Ac}-}}\right)\right] \end{array}$$

The microbial growth starts with a lag phase of ca. 40 h being represented by a slow increase of current density (*j*) and biofilm thickness (*L*_Biofilm_) for all anode potentials (Figures 2B,C). After an exponential increase of *j* and *L*_Biofilm_, *j* peaks and decreases due to acetate depletion (Figure 2E). As maintenance (i.e., metabolic costs for sustaining biomass) exceeds microbial energy harvest, additional energy for sustaining biomass has to be delivered by endogenous respiration leading to shrinkage of *L*_Biofilm_ (Wanner et al., 2006). At lower potentials (*E*_A_ ≤ 0.1 V), the increase of *j* and *L*_Biofilm_ lag behind that at more positive potentials (*E*_A_ ≥ 0.2 V) indicating restricted energy harvest (Figures 2B,C). The thermodynamic frame of *Geobacter* spp. biofilm is defined by acetate concentration, bicarbonate concentration, pH, and NAD^+^/NADH ratio (see Equation 9). The latter is kinetically affected by the anode potential. This can be quantified with the Butler-Volmer equation (Equation 10)^5^. This fundamental electrochemical equation describes the dependency of current from the applied overpotential (i.e., potential difference between active redox species and electrode) and according to the Butler-Volmer equation, a low potential difference between anode and cytochromes ($E_{\text{A}}-E^{\text{f}}$) results in a slow direct EET rate (i.e., current).

(10) $$j=j_{0}\left\{\exp\left[\frac{\alpha zF}{RT}\left(E_{\text{A}}-E^{\text{f}}\right)\right]-\text{exp}\left[\frac{(1-\alpha )zF}{RT}\left(E_{\text{A}}-E^{\text{f}}\right)\right]\right\}$$

When a low anode potential is applied, more cytochromes remain in the reduced state and the electron transfer from NADH to these is impeded resulting in a higher concentration thereof. In turn, this backlog of electrons in the NAD^+^/NADH pool impacts catabolic acetate oxidation as availability of NAD^+^ is limited (Figure 2A). The shift of NAD^+^/NADH ratio to lower values leads to an only partial exploitation of the thermodynamic frame and a restricted energy harvest (Δ*G*_cat_, see Equation 9). At lower potentials (*E*_A_ ≤ 0.1 V), the direct EET rate is slow compared to the acetate oxidation rate leading to high concentrations of NADH and reduced cytochromes (Supplementary Figures S2A,B). Only when the direct EET rate is sufficiently high (*E*_A_ ≥ 0.2 V), a high NAD^+^/NADH ratio is maintained (Supplementary Figure S2A) resulting in full exploitation of the thermodynamic frame. The degree of exploitation of the thermodynamic frame can be illustrated by means of microbial energy harvest (*U*_Harvest_, Equation 11)^6^. In the model, it represents the energy harvest from acetate oxidation integrated over biofilm thickness and time. Thereby, acetate gradients across biofilm thickness and during time are considered for calculations (Equation 11).

(11) $$\begin{array}{l} U_{\text{Harvest}}=\int_{0}^{t}\left(\int_{0}^{L_{\text{Biofilm}}}\Delta_{\text{R}}G_{\text{Cat}}r_{\text{Ac}-}\text{d}x\right){\text{d}\text{t}A}_{\text{A}} \end{array}$$

In the exponential phase, *S*_M_ is lower for *Geobacter* spp. biofilms at *E*_A_ ≤ 0.1 V compared to more positive anode potentials (*E*_A_ ≥ 0.2 V). Even when acetate is completely consumed, *S*_M_ remains clearly lower for *E*_A_ ≤ 0.1 V (Figure 2D). As described above, the decreased *S*_M_ is not immediately caused by a thermodynamic effect (i.e., a smaller energy difference between acetate and anode) but rather by EET kinetics interfering exploitation of the thermodynamic frame via a decreased NAD^+^/NADH ratio. When higher potentials are applied (*E*_A_ ≥ 0.2 V), the direct EET rate increases according to the Butler-Volmer equation (Equation 10) resulting in more oxidized cytochromes and a high NAD^+^/NADH ratio throughout the simulations (Supplementary Figures S2A,B). Therefore, the thermodynamic frame is fully exploited (i.e., harvested Δ_R_*G*_cat_ is maximized) leading to a maximized *S*_M_ (Figure 2D). At *E*_A_ = 0.2 V, *S*_M_ saturates and does not substantially further increase with more positive potentials (Figure 2D) demonstrating that the NAD^+^/NADH ratio is already sufficiently high and the thermodynamic frame is fully exploited. As the harvested energy does not further increase, *j* and *L*_Biofilm_ saturate at *E*_A_ = 0.2 V, too (Figures 2B,C). The saturation of *S*_M_ for simulation cases with *E*_A_ ≥ 0.2 V demonstrates the limited impact of the anode potential on the exploitation of the thermodynamic frame and energy-harvesting processes.

## Discussion

It is a general consensus that periplasm and the outer membrane are not energized compartments (Nicholls and Ferguson, 2013), hence a considerable number of *Geobacter* spp. cytochromes (i.e., periplasmic and outer membrane cytochromes) used for direct EET do not contribute to energy harvest (Bird et al., 2011). As a deduced consequence discussed in this article, the anode potential does not immediately influence energy harvest of EAM as the electron transfer step to the TEA is not part of the thermodynamic frame. Instead, thermodynamic assessments have to be conducted by considering the energy difference between substrate and an intracellular electron carrier.

For acetate oxidizing *Geobacter* spp. biofilm anodes, the impact of the anode potential on microbial energy harvest is solely a kinetic effect of the EET rate on exploitation of the thermodynamic frame. This was demonstrated by applying a previously developed model for direct EET. By increasing the potential difference between anode and cytochromes, the direct EET rate increases and cytochromes as well as NADH in the intracellular pools are faster oxidized (Supplementary Figures S2A,B). The resulting higher ratio of NAD^+^/NADH allows full exploitation of the thermodynamic frame defined by catabolic reactants (Equation 9). In the model, microbial energy harvest saturates at *E*_A_ ≥ 0.2 V and a further increase of the anode potential does not lead to higher microbial energy harvest. This often neglected bioenergetics fundamental was exemplified with a modeled *Geobacter* spp. biofilm anode oxidizing acetate but can be certainly expanded to other members of the diverse world of EAM (Koch and Harnisch, 2016).

The limited utilization of the provided redox potential from insoluble TEA for energy harvest represents a thermodynamic disadvantage for EAM compared to microorganisms respiring soluble TEA. In the light of the microbial window of opportunity or thermodynamic frame: the maximum available energy for EAM is smaller compared to the energy that can be harvested when a soluble TEA is utilized. At the same time, direct EET still enables EAM to grow in limited natural environments and the cytochrome system is able to store electrons representing an additional advantageous capacity for surviving in energy-limited habitats (Esteve-Núñez et al., 2008; Bonanni et al., 2012; Deng et al., 2018). Furthermore, *Geobacter* spp. can express different inner membrane cytochromes depending on the provided redox potential representing an opportunity to optimize energy harvest (Wagner et al., 2010; Levar et al., 2014, 2017;Zacharoff et al., 2016).

Processes like the microbial electrochemical Peltier heat, the redox-Bohr effect, and substrate-level phosphorylation ought to be considered for energetic assessments as they certainly will influence the energy balance of EAM. For instance, substrate-level phosphorylation represents the primary energy source for anaerobically growing *Shewanella oneidensis* MR-1 (Hunt et al., 2010). Although it is generally assumed that *Geobacter* spp. do not perform substrate-level phosphorylation (Galushko and Schink, 2000; Mahadevan et al., 2006), the data fundament on EAM using an anode as TEA is rather scarce and hence calls for deeper insights.

A comprehensive analysis of the energy balance of EAM is challenging and needs adapted thermodynamic approaches. Moreover, a correct perception of thermodynamics is urgently required for performing proficient research on EAM that in turn represents a necessity for a successful transfer to practice of MET.

## Author Contributions

All authors listed have made a substantial, direct and intellectual contribution to the work, and approved it for publication.

### Conflict of Interest Statement

The authors declare that the research was conducted in the absence of any commercial or financial relationships that could be construed as a potential conflict of interest.

## Footnotes

^1^*z* is number of transferred electrons and *F* is Faraday constant.

^2^1 mol L^−1^ of the respective reactants, 298.15 K, 101.325 kPa, pH = 7.

^3^*R* is universal gas constant, *T* is temperature, and $C_{\text{i}}^{0^{\prime }}$ is standard concentration of *i*-th reactant (1 mol L^−1^).

^4^All potential values in the article are provided vs. standard hydrogen electrode (SHE).

^5^*j*_0_ is exchange current density, and α is charge transfer coefficient.

^6^*A*_A_ is projected anode area.
