# Corrigendum: Spotlight on the Energy Harvest of Electroactive Microorganisms: The Impact of the Applied Anode Potential

**DOI:** 10.3389/fmicb.2019.02744

**Published:** 2019-11-28

**Authors:** Benjamin Korth, Falk Harnisch

**Affiliations:** Department of Environmental Microbiology, Helmholtz Centre for Environmental Research - UFZ, Leipzig, Germany

**Keywords:** electroactive microorganisms, extracellular electron transfer, microbial thermodynamics, microbial energy harvest, electron-transport chain, modeling

In the original article, Equation (11) that described the degree of exploitation of the thermodynamic frame by the microorganisms was misleading and has to be corrected for a more precise explanation. The symbol *C*_Ac−_ (acetate concentration) has to be replaced by *r*_Ac−_ (rate of acetate degradation). Therefore, the result of Equation (11) is microbial energy harvest (*U*_Harvest_) and not microbial action (*S*_M_).

A correction has been made to the section **Modeling the Energy Harvest of Electroactive Microorganisms**:

The degree of exploitation of the thermodynamic frame can be illustrated by means of microbial energy harvest (*U*_Harvest_, Equation 11)^6^. In the model, it represents the energy harvest from acetate oxidation integrated over biofilm thickness and time. Thereby, acetate gradients across biofilm thickness and during time are considered for calculations (Equation 11).

Equation (11) in the section **Modeling the Energy Harvest of Electroactive Microorganisms** was corrected:

(11) $$\begin{array}{l} U_{\text{Harvest}}=\int_{0}^{t}\left(\int_{0}^{L_{\text{Biofilm}}}\Delta_{\text{R}}G_{\text{Cat}}r_{\text{Ac}-}\text{d}x\right){\text{d}\text{t}A}_{\text{A}} \end{array}$$

Accordingly, Figure 2D and the legend of Figure 2 in the section **Modeling the Energy Harvest of Electroactive Microorganisms** were corrected and appear below:

**Figure 2:**
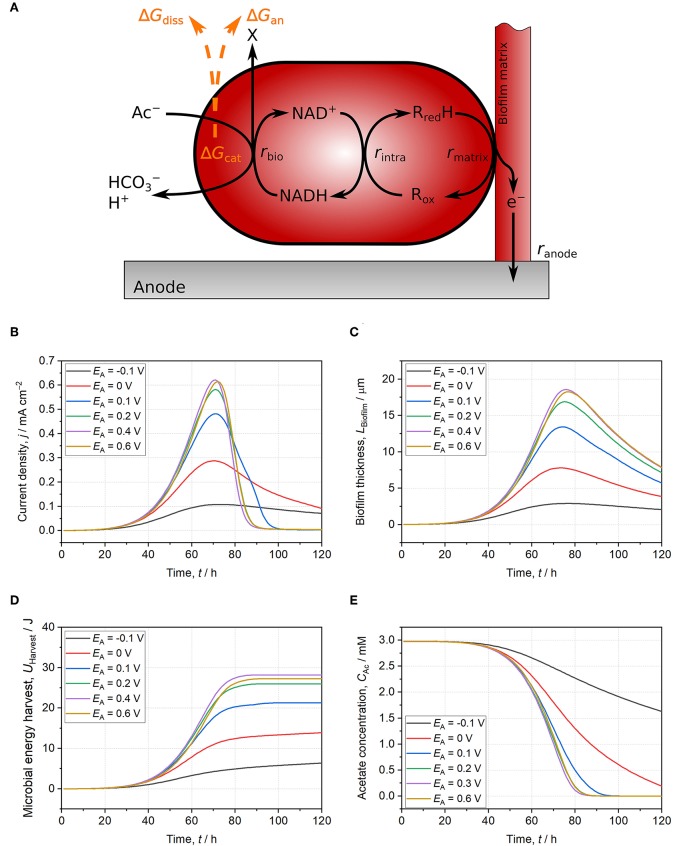
Schematic illustration of the used model and model results for *Geobacter* spp. biofilms growing on anodes set to −0.1 V (black line), 0 V (red line), 0.1 V (blue line), 0.2 V (green line), 0.4 V (purple line), and 0.6 V (yellow line). **(A)** Schematic model representation: Acetate oxidation is coupled to NAD^+^ reduction resulting in energy harvest (Δ*G*_cat_) subsequently used for the build-up of biomass (Δ*G*_an_) and for providing driving force for growth (Δ*G*_diss_). Electrons are then transferred to intracellular cytochromes and further to a conductive biofilm matrix. Finally, electrons are donated to the anode. All reactions occur at individually calculated rates (*r*_bio_, *r*_intra_, *r*_matrix_, *r*_anode_) (Korth et al., 2015). **(B)** Current density. **(C)** Biofilm thickness. **(D)** Microbial energy harvest. **(E)** Acetate concentration. With anode potentials ≤ 0.1 V, the thermodynamic frame defined by acetate, NAD^+^/NADH ratio, and other reactants is not fully exploited. Slow EET kinetics result in thermodynamically unfavorable reaction conditions for catabolic reaction (i.e., low NAD^+^/NADH ratio) leading to lower current density, biofilm thickness, and microbial energy harvest at comparable acetate consumption. For anode potentials ≥ 0.2 V, direct EET is not limiting catabolism and reaction conditions are thermodynamically improved. Consequently, current density, biofilm thickness, and microbial energy harvest do not further increase with higher potentials. The model is based on separated anodic compartment (volume = 250 mL, anode area = 10 cm^2^) and cathodic compartment via a membrane (membrane area = 10 cm^2^). Acetate concentration is 3 mM, phosphate buffer concentration is 50 mM, and initial pH is 6.95. Further model parameters are detailed in Supplementary Table S1 and Korth et al. (2015).

Additionally, the section **Discussion** was corrected:

In the model, microbial energy harvest saturates at *E*_A_ ≥ 0.2 V and a further increase of the anode potential does not lead to higher microbial energy harvest.

The authors apologize for this error and state that this does not change the scientific conclusions of the article in any way. The original article has been updated.

## Footnotes

^6^*A*_A_ is projected anode area.

## References

[B1] Korth B. Rosa L. F. M. Harnisch F. Picioreanu C. (2015). A framework for modeling electroactive microbial biofilms performing direct electron transfer. Bioelectrochemistry 106, 194–206. 10.1016/j.bioelechem.2015.03.010 25921352

