# Supplementary material for: Spotlight on the Energy Harvest of Electroactive Microorganisms: The Impact of the Applied Anode Potential
Source: Front Microbiol. 2019 Jun 26;10:1352. doi: 10.3389/fmicb.2019.01352 (PMC6606774; doi:10.3389/fmicb.2019.01352)
Supplement: Supplementary file 1 [file Data_Sheet_1.docx]

Supplementary Material

**Spotlight on the energy harvest of electroactive microorganisms: The impact of the applied anode potential**

**Benjamin Korth^*^ and Falk Harnisch^*^**

Department of Environmental Microbiology, Helmholtz Centre for Environmental Research - UFZ, Leipzig, Germany

Corresponding authors: [benjamin.korth@ufz.de](mailto:benjamin.korth@ufz.de); [falk.harnisch@ufz.de](mailto:falk.harnisch@ufz.de)

# Supplementary Table S1

| **Parameter description** | **Symbol** | **Value** | **Unit** |
| --- | --- | --- | --- |
| Concentration NAD^+^/NADH | $C_{NAD+/NADH}$ | 0.003 | mol L^−1^ |
| Concentration oxidized/reduced redox centers^[[1]](#footnote-1)^ | $C_{Rox/Rred}$ | 0.150 | mol L^−1^ |
| Initial biofilm thickness | $L_{Biofilm,0}$ | 0.01 | µm |
| Temperature | $T$ | 308.15 | K |
| Forward rate for the intracellular electron transfer from NADH to redox centers | $k_{f,m}$ | 250 | m^9^ mol^−3^ s^−1^ |
| Reverse rate constant for the intracellular electron transfer from redox centers to NAD^+^ | $k_{r,m}$ | 1$\times$10^−10^ | m^6^ mol^−2^ s^−1^ |

# Supplementary Figure S2





Figure S2 Model results for *Geobacter* spp. biofilm growing on an anode set to −0.1 V (black line), 0 V (red line), 0.1 V (blue line), 0.2 V (green line), 0.4 V (purple line), and 0.6 V (yellow line) **(A)** NAD^+^/NADH ratio. **(B)** Ratio of oxidized redox centers and reduced redox centers. Concentration values of NAD^+^/NADH and oxidized/reduced redox centers were integrated over the whole biofilm thickness. During the simulation, the ratio of oxidized/reduced redox centers decrease according to catabolic activity and increase again as acetate is depleted. The backlog of electrons is transmitted to the NAD^+^/NADH pool. With *E*_A_ ≤ 0.1 V, the NAD^+^/NADH ratio decreases, resulting in a poorer exploitation of the thermodynamic frame. During simulations with *E*_A_ ≥ 0.2 V, higher NAD^+^/NADH ratios, and thus full exploitation of the thermodynamic frame are maintained.

1. The term “cytochrome” describes a protein moiety containing one or several hemes as redox-active cofactors and each heme can receive or donate one electron at the same time. The term “redox center” as it is used in the model designates a moiety that can receive and donate one electron at the time. Thus a redox center can represent a heme group of a cytochrome, whereas, *e.g.*, a menaquinone is represented by two redox centers, as it is able to bind two electrons. Thus, “redox center” and “cytochrome” are strictly speaking interchangeable terms. For *Geobacter* spp. the main redox centers are cytochromes and hence for a better understanding only the terminus cytochrome is used in the main manuscript and the term redox center is used in the SI when referring specifically to the model. [↑](#footnote-ref-1)
